# Targeting Bacterial Gyrase with Cystobactamid, Fluoroquinolone, and Aminocoumarin Antibiotics Induces Distinct Molecular Signatures in Pseudomonas aeruginosa

**DOI:** 10.1128/mSystems.00610-21

**Published:** 2021-07-13

**Authors:** Raimo Franke, Heike Overwin, Susanne Häussler, Mark Brönstrup

**Affiliations:** a Department of Chemical Biology, Helmholtz Centre for Infection Research, Braunschweig, Germany; b Institute of Molecular Bacteriology, Twincore, Centre for Clinical and Experimental Infection Research, Hannover, Germany; c Department of Molecular Bacteriology, Helmholtz Centre for Infection Research, Braunschweig, Germany; d German Centre for Infection Research (DZIF), Braunschweig, Germany; University of California, Berkeley

**Keywords:** antibiotics, *Pseudomonas aeruginosa*, mode of action, gyrase, metabolomics, RNA sequencing, DNA gyrase

## Abstract

The design of novel antibiotics relies on a profound understanding of their mechanism of action. While it has been shown that cellular effects of antibiotics cluster according to their molecular targets, we investigated whether compounds binding to different sites of the same target can be differentiated by their transcriptome or metabolome signatures. The effects of three fluoroquinolones, two aminocoumarins, and two cystobactamids, all inhibiting bacterial gyrase, on Pseudomonas aeruginosa at subinhibitory concentrations could be distinguished clearly by RNA sequencing as well as metabolomics. We observed a strong (2.8- to 212-fold) induction of autolysis-triggering pyocins in all gyrase inhibitors, which correlated with extracellular DNA (eDNA) release. Gyrase B-binding aminocoumarins induced the most pronounced changes, including a strong downregulation of phenazine and rhamnolipid virulence factors. Cystobactamids led to a downregulation of a glucose catabolism pathway. The study implies that clustering cellular mechanisms of action according to the primary target needs to take class-dependent variances into account.

**IMPORTANCE** Novel antibiotics are urgently needed to tackle the growing worldwide problem of antimicrobial resistance. Bacterial pathogens possess few privileged targets for a successful therapy: the majority of existing antibiotics as well as current candidates in development target the complex bacterial machinery for cell wall synthesis, protein synthesis, or DNA replication. An important mechanistic question addressed by this study is whether inhibiting such a complex target at different sites with different compounds has similar or differentiated cellular consequences. Using transcriptomics and metabolomics, we demonstrate that three different classes of gyrase inhibitors can be distinguished by their molecular signatures in P. aeruginosa. We describe the cellular effects of a promising, recently identified gyrase inhibitor class, the cystobactamids, in comparison to those of the established gyrase A-binding fluoroquinolones and the gyrase B-binding aminocoumarins. The study results have implications for mode-of-action discovery approaches based on target-specific reference compounds, as they highlight the intraclass variability of cellular compound effects.

## INTRODUCTION

The rise of antimicrobial resistance (AMR) has been identified as a serious threat for global health (1). The number of novel antibiotics in clinical development to fight AMR is small, and most of them represent improved versions of established drug classes. To acknowledge “innovation,” antibiotic pipeline reports highlight those belonging to a new structural class and those with a new mechanism of action (2). However, the latter classification into new versus old is not clear-cut: for example, does an antibiotic that addresses a novel binding site on bacterial ribosomes and that causes a distinct arrest of translation fall into “new” or “old”? A medically relevant criterion concerns the ability of the new agent to break resistance, whereas a more mechanistic perspective looks at overall cellular consequences of antibiotic treatments, e.g., on the cellular transcriptome or metabolome (3, 4). A series of recent elegant studies has demonstrated that antibiotics addressing different cellular targets induce differential responses on the metabolome or transcriptome (5–9). Moreover, the typical response pattern can serve to assign the mode of action of novel antibacterial compounds (6, 9). The underlying assumption behind such target-predicting methods, which are also widely applied for the study of eukaryotic cell biology (10, 11), is that the molecular signatures of bioactive compounds cluster according to the target, irrespective of their structure.

We wanted to probe this assumption and questioned whether or not antibiotics that bind to the same target but belong to different structural classes induced distinguishable molecular signatures. For this purpose, representatives of three classes of DNA gyrase inhibitors, the fluoroquinolones, the aminocoumarins, and the recently discovered cystobactamids, were selected and compared with respect to their metabolome and transcriptome effects on the opportunistic human pathogen Pseudomonas aeruginosa. The Gram-negative, opportunistic pathogen P. aeruginosa is a causative agent for nosocomial infections and for infections associated with cystic fibrosis, classified as a priority 1 pathogen by the WHO (12). P. aeruginosa shows substantial resistance to antibiotics by expression of multidrug efflux pumps and establishes infection by specific virulence factors, such as phenazines and rhamnolipids (13). The gyrase A-binding fluoroquinolones, in particular ciprofloxacin, constitute a standard treatment of P. aeruginosa infections (14). Subinhibitory concentrations of ciprofloxacin induce phenotypic alterations such as a reduction in swimming and swarming (14, 15) but also modulate the expression of hundreds of genes (14, 16, 17).

Recently, a novel class of gyrase inhibitors, the cystobactamids, were isolated from the soil myxobacterium *Cystobacter* sp. (18, 19). It could be shown in gyrase supercoiling assays and gyrase mutant assays that cystobactamids stabilize the gyrase cleavage complex, indicating that they are type II topoisomerase poisons (18). Cystobactamids engage the gyrase A subunit for target binding as well as the minor groove of the bound DNA through the right-hand side of the molecule (20). Medicinal chemistry efforts lead to cystobactamid analogs like CN-DM-861 and AR351, which showed improved activities against P. aeruginosa and other bacterial pathogens compared to those of the natural products (21, 22). As a third class, we included the aminocoumarins, which are competitive inhibitors of the ATP-binding site of the gyrase B subunit (23). Crystal structures revealed that the deoxy-sugar of the aminocoumarins overlaps the ATP-binding site and thereby inhibits the catalytic activity of the ATPase (24, 25). In contrast to cleavage-complex-stabilizing compounds or gyrase poisons, aminocoumarins do not cause DNA double-strand breaks (25).

While transcriptome effects of ciprofloxacin on P. aeruginosa have been reported before (15–17, 26), this study represents the first integrative and systematic investigation of the cellular effects associated with gyrase inhibition across three drug classes of gyrase inhibitors.

## RESULTS AND DISCUSSION

### Gyrase inhibitors at subinhibitory concentration induce class-specific large-scale changes in Pseudomonas aeruginosa PA14 transcriptome and metabolome.

**FIG 1:**
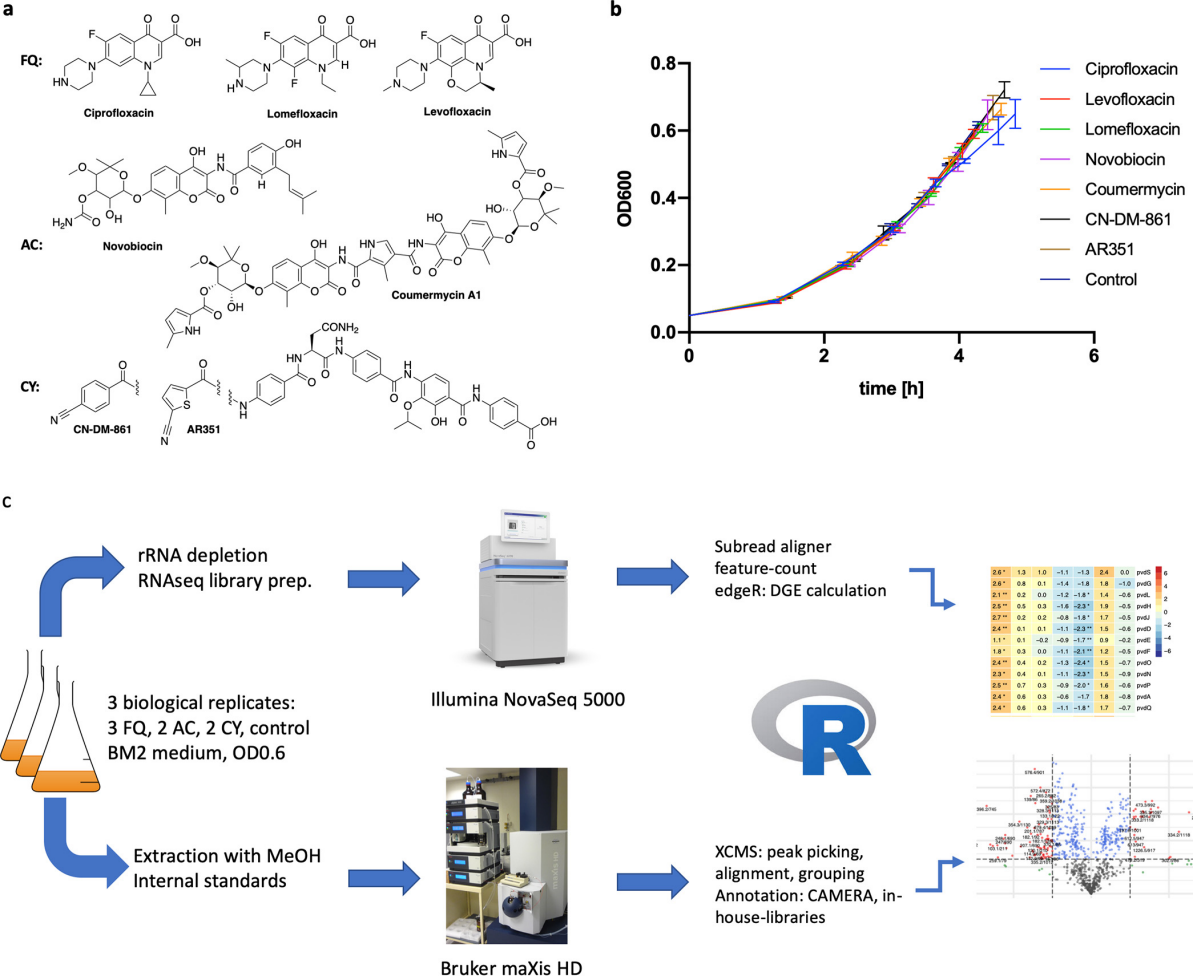
Study design. (a) Chemical structures and names of the antibiotics used in this study. The chemical class (FQ for fluoroquinolones, AC for aminocoumarins, CY for cystobactamids) is indicated to the left. (b) Growth curves of the P. aeruginosa PA14 cultures after treatment with antibiotics at IC_50_. Curves display the mean of biological triplicates, and the error bars show the standard deviation. (c) Multi-omics workflow used for this study.

In order to identify and compare interclass versus intraclass effects of gyrase inhibitors on the bacterial transcriptome and metabolome, the P. aeruginosa model strain PA14 was exposed to the three fluoroquinolones ciprofloxacin, levofloxacin, and lomefloxacin, the two aminocoumarins novobiocin and coumermycin A1, and the two cystobactamids CN-DM-861 and AR351 (Fig. 1a). To avoid that the observed effects simply reflected consequences of altered growth, subinhibitory concentrations were applied that did not impair growth until the target optical density (OD) of 0.6, corresponding to a mid-exponential phase, was reached. We selected the respective 50% inhibitory concentration (IC_50_) as the sub-MIC of the compounds (Table 1), as these conditions did not impair growth but allowed to capture the transcriptional and metabolic response to the antibiotic challenge after several hours of exposure. The obtained growth curves were identical, and a minimal retardation of growth for ciprofloxacin-treated cultures was nonsignificant (Fig. 1b).

**TABLE 1:** Antibacterial activities and number of regulated transcripts and metabolite features of gyrase inhibitors

Antibiotic	Class	MIC (μg/ml)	IC_50_ (μg/ml)	No. genes upregulated^*a*^	No. genes downregulated^*a*^	No. features upregulated^*a*^	No. features downregulated^*a*^
Ciprofloxacin	Fluoroquinolone	0.1	0.06	343	179	63	87
Levofloxacin	Fluoroquinolone	0.2	0.1	172	45	20	77
Lomefloxacin	Fluoroquinolone	1.2	0.3	203	71	32	83
Novobiocin	Aminocoumarin	388	213	277	444	48	176
Coumermycin A1	Aminocoumarin	5.3	3.8	512	825	123	165
CN-DM-861	Cystobactamid	3.9	1.8	163	14	60	10
AR351	Cystobactamid	2.4	2.2	86	8	154	41

a Differentially expressed genes (fold change > 2, FDR < 0.05), differential abundance of metabolite features (fold change > 1.5, *P* < 0.05).

The metabolomes and transcriptomes were detected by untargeted liquid chromatography coupled to (tandem) mass spectrometry (LC-MS/MS) and RNA sequencing, respectively, according to a workflow depicted in Fig. 1c. RNA and metabolites were isolated from the same cultures at the target OD to ensure correspondence of transcriptome and metabolome. Metabolites were extracted using 80% methanol, separated by reverse-phase chromatography, and analyzed using electrospray ionization-high-resolution time-of-flight mass spectrometry (ESI-QTOF-MS). The extraction protocol was a compromise in order to access a fraction of primary metabolites and also to cover the richness of P. aeruginosa’s secondary metabolome. The secondary metabolome was of particular interest to us because of the emerging understanding that subinhibitory concentrations of antibiotics function as signals to activate bacterial secondary metabolism (27). Data preprocessing led to a peaktable with 836 features. After deisotoping and removal of features that stem from internal standards or the applied gyrase inhibitors, 657 features remained, of which 123 could be annotated (Tables S1 and S3).

10.1128/mSystems.00610-21.2 TABLE S1 Annotated metabolites from the untargeted metabolomics analysis. RT, retention time; annotation, annotation of the ion, unless stated otherwise the [M + H]^+^ ion; metabolite, metabolite the ion belongs to; ID method, identification method; MS, identification by MS1 exact mass; RT, retention time; MS2, matching of tandem MS data; ID level, level of confidence of identification according to the Metabolomics Standards Initiative of the Metabolomics Society. Download Table S1, DOCX file, 0.02 MB. Copyright © 2021 Franke et al. 2021 Franke et al. https://creativecommons.org/licenses/by/4.0/ This content is distributed under the terms of the Creative Commons Attribution 4.0 International license.

10.1128/mSystems.00610-21.4 TABLE S3 Results of the untargeted metabolomics analysis (provided in a separate Excel file). mz, m/z; rt, retention time in s; anno, annotation of the ion; metabolite, metabolite the ion belongs to; main_peak, peak with highest intensity; ID by, identification by MS1 (exact mass), RT (retention time), MS2 (matching of tandem MS data); ID level, level of confidence of identification according to the Metabolomics Standards Initiative of the Metabolomics Society. Download Table S3, XLSX file, 0.5 MB. Copyright © 2021 Franke et al. 2021 Franke et al. https://creativecommons.org/licenses/by/4.0/ This content is distributed under the terms of the Creative Commons Attribution 4.0 International license.

After RNA was isolated and purified, the constructed cDNA library was sequenced on a NovaSeq device (2 × 50 bp), followed by short read-alignment, count estimation, and differential expression analysis.

**FIG 2:**
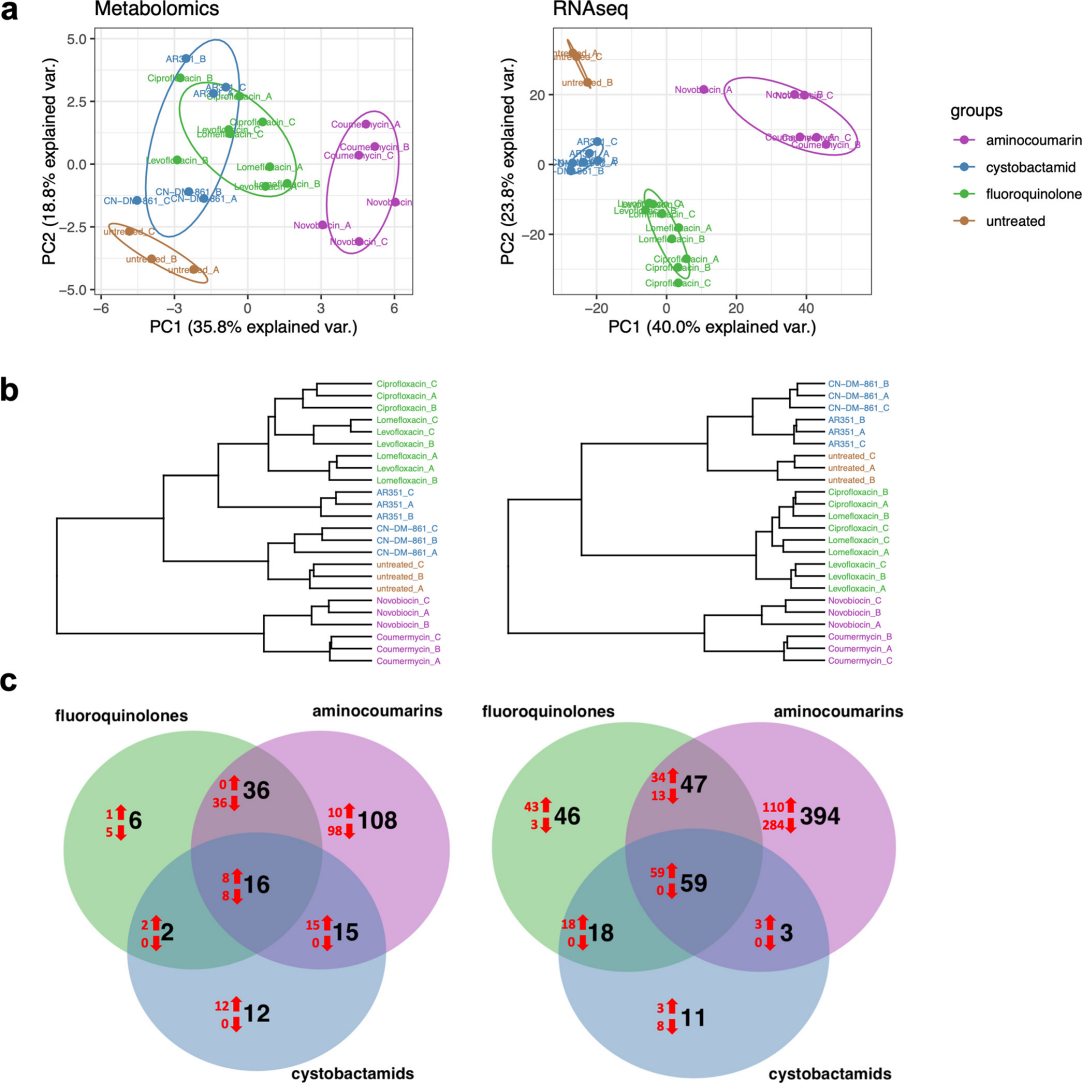
Global outcome of transcriptome and metabolome analysis. (a) Principal-component analysis (PCA) scores plots. Left, metabolome, calculated from all scaled and centered metabolite features; right: transcriptome, centered log CPM expression data of all genes. (b) Hierarchical clustering using Ward’s linkage criterion. Left, metabolome, Euclidean distances calculated from all normalized and scaled metabolic features; right, transcriptome, Euclidean distance calculated from log CPM expression values. (c) Venn diagram displaying the numbers of differentially abundant metabolic features and transcripts for each class of gyrase inhibitors compared to untreated controls (total numbers in black, numbers in red show up-/downregulated features or genes). Left, differentially abundant metabolic features (log_2_fold change [FC] > log_2_[1.5] or log_2_FC < −log_2_[1.5] and *P* < 0.05); right, differentially expressed genes (log_2_FC > 1 or log_2_FC < −1 and FDR < 0.05).

We first performed a principal-component analysis (PCA) and a hierarchical clustering on both the transcriptome and metabolome data. All scaled and centered features were used for the PCA of the metabolomics data, and centered log_2_-transformed counts per million (log CPM) data of all 5,979 transcripts were used for the PCA of the transcriptomics data. The PCA score plots of transcripts showed a complete separation of the fluoroquinolones, aminocoumarins, cystobactamids, and the untreated controls for all biological replicates (Fig. 2a). In the PCA score plot of the metabolite features, the classes were also separated, but the 95% confidence ellipses were more spread out, and the cystobactamids partially overlapped the fluoroquinolones. For hierarchical cluster analysis, all pairwise Euclidean distances using the normalized and scaled metabolite features (Fig. 2b, left) and log CPM values of the transcripts (Fig. 2b, right) were calculated. For the RNA-Seq data, three main clusters were formed. The aminocoumarins were grouped in one cluster, with the biological replicates in closest proximity that had the largest distance to all others. The fluoroquinolones spanned a second cluster, with levofloxacin replicates in one subcluster and ciprofloxacin and lomefloxacin in another. The third cluster was split into a cystobactamid subcluster and into a subcluster with the untreated control replicates, indicating that cystobactamids exerted the smallest effect on PA14 transcriptome and showed the highest similarities to the untreated controls. The clustering of the metabolomics data gave a similar result, with the clearest separation of the aminocoumarin cluster from the others. Also, the fluoroquinolones spanned a distinct subcluster. Cystobactamids split into two subclusters, with AR351 replicates in closer proximity to the fluoroquinolones, whereas the CN-DM-861 cluster was grouped closer to the untreated control. Overall, fluoroquinolones, aminocoumarins, and cystobactamids have distinct effects on the transcriptome and metabolome of PA14 and can be distinguished according to their class; the gyrase B-binding aminocoumarins were distant from the gyrase A-binding fluoroquinolones and cystobactamids. The differences cannot be traced back to a technical variance of the method, because both biological replicates of a given compound as well as different congeners of a compound class (i.e., ciprofloxacin, levofloxacin, and lomefloxacin) were more similar to each other than to members of other classes. While a complete separation was achieved for the transcriptome data, metabolome data had a broader spread. This can be explained with the higher experimental variability of the metabolomics extraction protocol. In addition, the expression values of all 5,979 transcripts were assessed, whereas only the midpolar fraction with an emphasis on secondary metabolites of the PA14 metabolome was sampled, thus obscuring a potential class separation by nonrepresented metabolites undetected.

To pinpoint the transcripts and metabolites that contribute to class separation, their relative abundances, expressed as fold changes in comparison to those of the untreated controls, and the associated *P* values were calculated for all analytes. Cutoffs for fold change of 2 for the transcripts and of 1.5 for the metabolite features were applied. The number of transcripts and metabolite features that are regulated by all members of one inhibitor class are shown in Table 2, and those that are jointly regulated by all members of two or all three classes are depicted in Venn diagrams (Fig. 2c). The degree of regulation found for transcriptome and that found for metabolome correlate well. The numbers of significantly regulated transcripts and differentially abundant metabolites differed strikingly between the antibiotic classes (Tables 1 and 2 and Tables S2 and S3): whereas cystobactamids affected the smallest number of transcripts and metabolic features, the aminocoumarins induced differential effects in the highest number of genes and metabolic features. Particularly regarding downregulation of transcripts and feature levels, the aminocoumarins had by far the most pronounced effect. The fluoroquinolones in comparison also induced upregulation of 154 genes (intersection of the three fluoroquinolones used) but had fewer downregulating effects (Tables 1 and 2). Aminocoumarins also had the highest number of differentially regulated transcripts and metabolic features that are not shared with the two other classes. The Venn diagrams in Fig. 2c show the intersections of the transcripts and metabolic features that are jointly affected by two or three classes of gyrase inhibitors. The pool of transcripts and metabolic features used to construct the Venn diagrams was created by taking the intraclass intersections of regulated transcripts and differentially abundant metabolic features (i.e., regulated transcripts/features shared by all compounds of a class). Overall, the numbers of jointly regulated transcripts and metabolite features (59 and 21, respectively) are far smaller than the numbers of analytes affected by only one (up to 394 transcripts and up to 108 metabolite features) or two classes (up to 47 transcripts and up to 36 metabolite features). Thus, a striking result of this summary analysis is that the three classes of gyrase inhibitors have widely differing (rather than similar) imprints on PA14 transcriptome and metabolome with respect to the cutoff criteria. In the following section, we go through the most striking changes that are caused by gyrase inhibitor perturbation.

**TABLE 2:** Number of regulated transcripts and metabolite features shared by all members of a gyrase inhibitor class

Class	No. genes upregulated^*a*^	No. genes downregulated^*a*^	No. features upregulated^*a*^	No. features downregulated^*a*^
Fluoroquinolones	154	16	11	49
Aminocoumarins	206	297	33	142
Cystobactamids	83	8	37	8

a Differentially expressed genes (fold change > 2, FDR < 0.05), differential abundance of metabolite features (fold change > 1.5, *P* < 0.05).

10.1128/mSystems.00610-21.3 TABLE S2 Results of the RNA-Seq analysis (provided in a separate Excel file). ID, PA14 locus tag; PAO1_ID, PAO1 locus tag; name, gene name; Product_Name, name of the corresponding protein; GO_BP, gene ontology biological process annotation; KEGG_Pathway, annotation with metabolic pathway from the KEGG database; PseudoCAP_Pathway, pathway annotation by the Pseudomonas Community Annotation Project (PseudoCAP). Download Table S2, XLSX file, 1.5 MB. Copyright © 2021 Franke et al. 2021 Franke et al. https://creativecommons.org/licenses/by/4.0/ This content is distributed under the terms of the Creative Commons Attribution 4.0 International license.

### Gyrase inhibitors of all classes induce SOS responses.

**FIG 3:**
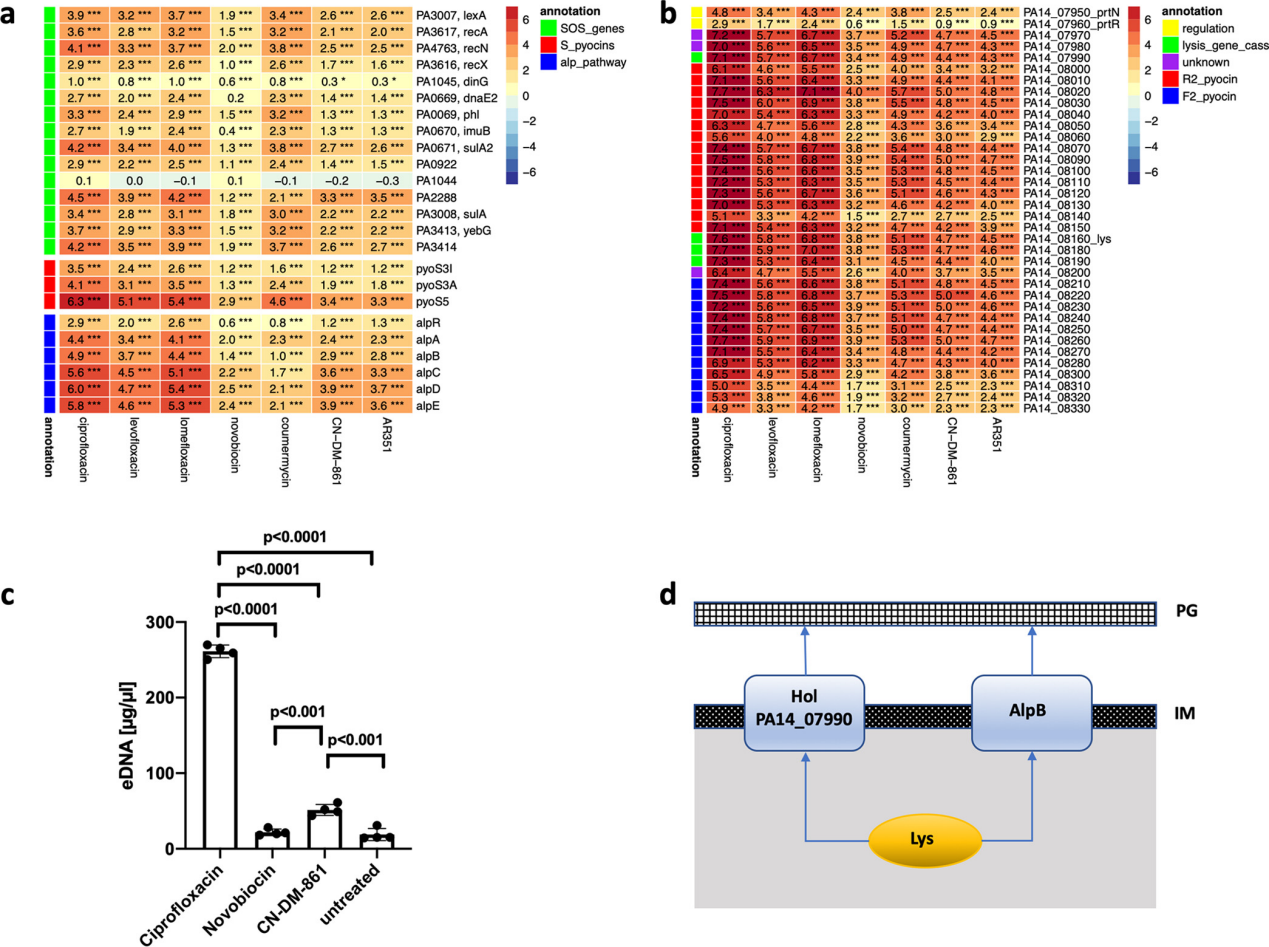
(a) Heatmap of differentially expressed genes of the SOS regulon, as defined by Cirz et al. (16), heatshock and chaperone genes, S-pyocins, and the Alp cell lysis pathway. PAO1 locus tags were mapped to PA14 locus tags where necessary. Numbers display the log_2_ fold change (log_2_FC) in comparison to the untreated control, which is also translated into the color scale. Number of asterisks denotes FDR thresholds: ***, 0.001; **, 0.01 *, 0.05. (b) Heatmap of differentially expressed genes of the prophage/RF-pyocin region. (c) Quantification of eDNA from planktonic cultures of PA14 at OD of 0.6, treated with ciprofloxacin (*n* = 4), novobiocin (*n* = 4), CN-DM-861 (*n* = 4), and buffer control (*n* = 4). The *P* values were determined by ordinary one-way analysis of variance (ANOVA) with Bonferroni’s multiple-comparison test (F = 1,047.0; df = 15). Error bars show standard deviation. (d) Scheme showing endolysin-mediated autolysis in P. aeruginosa adapted from reference 13.

Gyrase poisoning and the resulting DNA double-strand breaks according to previous studies (28) induce the SOS response, which is characterized by RecA-mediated LexA-induced gene expression. Because the LexA regulon of P. aeruginosa was first described for PAO1 (16), the PAO1 locus IDs were mapped to the PA14 genome using the BACTOME annotator (29). All gyrase inhibitors investigated in this study induced upregulation of the LexA regulon, albeit to different extents (Fig. 3a). The strongest induction was found for ciprofloxacin, followed by the other fluoroquinolones, whereas the cystobactamids had weaker effects. Surprisingly, the LexA regulon was also induced by the aminocoumarins, although they do not directly cause DNA double-strand breaks. A possible explanation is that DNA damage is caused not only by gyrase A poisoning but also through the action of reactive oxygen species (ROS). In fact, Collins and coworkers have shown that bactericidal antibiotics induce hydroxyl radical formation through an oxidative damage pathway, which then triggers RecA and the DNA damage response (30). The presence of ROS triggers cellular protective responses to oxidative modifications of proteins (e.g., carbonylation) (31). We indeed observed an upregulation of chaperones and heat shock proteins indicating protein damage (Fig. 3a). Thus, we speculate that the aminocoumarins elicit a strong protective response to ROS via upregulation of heat shock proteins and chaperones and a subsequent RecA-mediated SOS induction without causing direct DNA damage.

### Strong upregulation of pyocin regions and Alp pathway and detection of eDNA verify self-cell lysis in response to gyrase inhibitor treatment.

The most pronounced upregulation of gene expression as a consequence of gyrase inhibitor treatment could be mapped to the so-called prophage R/F-pyocin region of PA14. The effect was strongest for fluoroquinolones; in particular, ciprofloxacin induced up to 212-fold changes in transcript abundance (log_2_ fold change of 7.73) (Fig. 3b).

The upregulation of the R/F-pyocin region within the PAO1 genome was described in response to the treatment with hydrogen peroxide as a consequence of oxidative stress (32). It was also observed as a consequence of ciprofloxacin treatment of P. aeruginosa strains PAO1 and PAK (26, 33). We mapped the PAO1 locus IDs to orthologs in the PA14 genome and found exactly the same region to be affected by each gyrase inhibitor used in this study.

Pyocins are protein toxins that are used in interspecies competitions against the same or closely related strains. P. aeruginosa produces three types of pyocins: The S-type pyocin is a colicin-like protein, and the R- and F-type pyocins are derived from phage tails and are evolutionary specialized as bacteriocins (13). It has been shown that clinical isolates of P. aeruginosa produce distinct ranges of pyocins, which influence biofilm formation and the strain composition in the cystic fibrosis (CF) lung (34) and thus clinical outcome. The R/F-pyocin region is regulated by *prtN*, a positive regulator of pyocin production under the control of the *prtR* repressor. RecA causes the autocleavage of PrtR, leading to the expression of *prtN* and upregulation of expression of pyocins (35), and thereby links the SOS response directly to pyocin induction.

In fact, there is a qualitative correlation between the strength of SOS response and pyocin production by the different gyrase inhibitors in this study. Novobiocin treatment causes the weakest SOS induction and also the weakest pyocin induction, whereas ciprofloxacin is the strongest inducer for both processes.

Within the R/F-pyocin region, a putative holin protein (PA14_07990) and the endolysin Lys (PA14_08160) are encoded. According to Turnbull et al., Lys crosses the inner membrane via holin proteins and degrades the peptidoglycan cell wall, which eventually leads to cell lysis (36) (Fig. 3d). Self-lysis is also induced by the holin protein AlpB through the Alp pathway (37). Cleavage of the regulator AlpR leads to derepression of the *alpA* gene, which encodes a positive regulator that activates the expression of the *alpBCDE* lysis cassette (37). The Alp pathway contributes to the lethality for the individual cell, although the whole population might benefit by release of eDNA and biofilm formation.

We found that both *lys* and the Alp pathway were consistently upregulated, again with the fluoroquinolones eliciting the strongest effects, followed by the cystobactamids, whereas the aminocoumarins induced the smallest fold changes.

The autolysis of a fraction of the P. aeruginosa population should lead to the release of DNA into the medium. We therefore investigated whether the induction of the RF-pyocin region correlated with the amount of released extracellular DNA (eDNA). For this purpose, eDNA was quantified following a protocol by Allesen-Holm et al. using the same cultivation conditions as for the omics experiments (38), with one representative of each gyrase inhibitor class (Fig. 3c). For ciprofloxacin, the eDNA concentration of 261.28 μg/μl released to the medium exceeded the control value of 18.86 μg/μl by far (*P* < 0.0001). Also, the treatment with the cystobactamid CN-DM-861 led to a significant increase of eDNA (51.48 μg/μl, *P* < 0.001). In contrast, novobiocin induced no statistically significant difference compared to the control. We also tested for association between the expression of the endolysin *lys* in CPM and the amount of eDNA detected in μg/μl and obtained a Spearman rank correlation coefficient ρ of 1 (*P* = 0.083), indicating a strong positive correlation (Fig. S1). This is a clear hint that indeed the postulated mechanism of endolysin/holin upregulation leads to degradation of the peptidoglycan layer, destabilization of the cell wall, and finally autolysis of the bacterial cell. Self-lysis may contribute substantially to the efficacy of gyrase inhibitors, irrespective of their class.

10.1128/mSystems.00610-21.1 FIG S1 Regression line and confidence interval displaying the Spearman correlation of *lys* expression and the amount of detected eDNA. Spearman’s rho statistic is used to estimate a rank-based measure of association. For Spearman’s test, *P* values were computed using algorithm AS 89. Download FIG S1, TIF file, 2.5 MB. Copyright © 2021 Franke et al. 2021 Franke et al. https://creativecommons.org/licenses/by/4.0/ This content is distributed under the terms of the Creative Commons Attribution 4.0 International license.

### Aminocoumarins induce a strong downregulation of virulence factors.

The pathogenicity of P. aeruginosa is strongly mediated by low-molecular-weight virulence factors, including the phenazines, the rhamnolipids, and quorum-sensing mediators (39). The phenazines are redox-active heterocycles that contribute to pathogenesis by subjecting host cells to harmful oxidative stress (40). Two almost identical operons (*phz1* and *phz2*) code for the phenazine biosynthesis pathway, which produces phenzine-1-carboxylic acid as the precursor for all other phenazines, that is further converted to pyocyanin by PhzM and PhzS (41).

**FIG 4:**
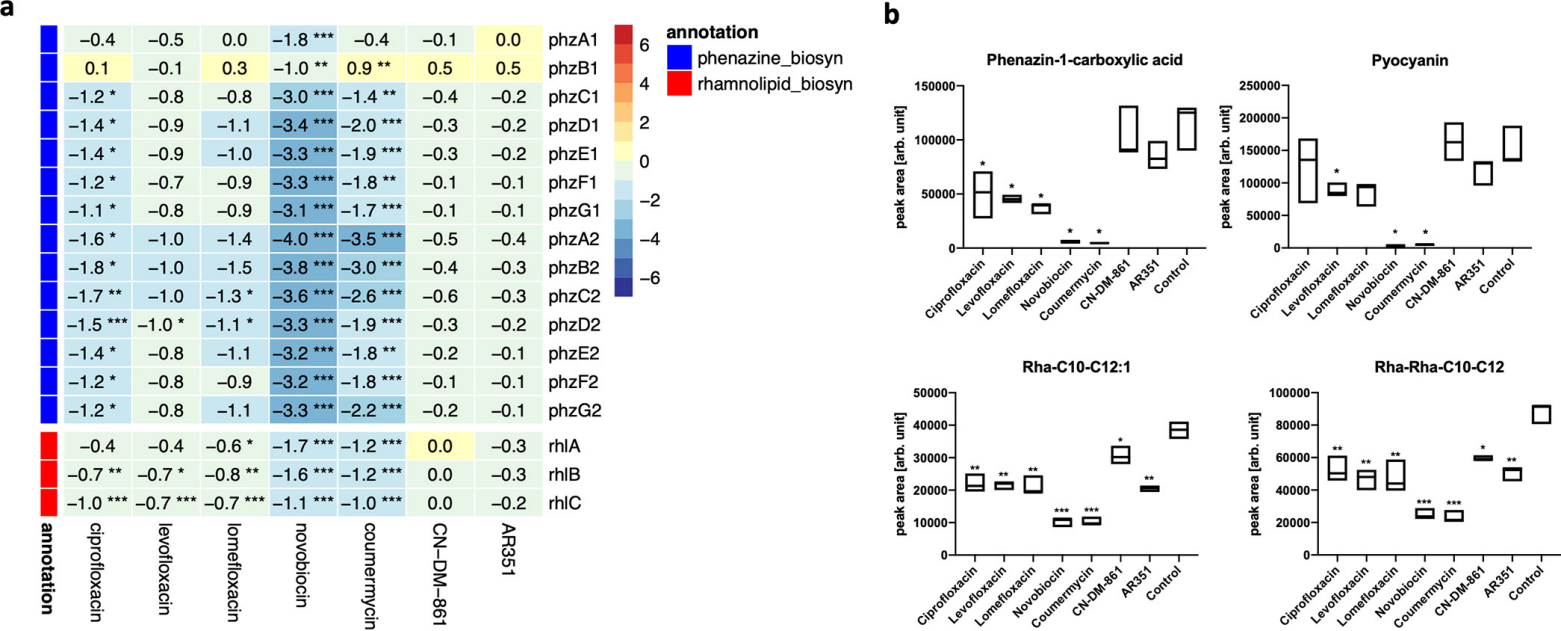
(a) Heatmap displaying log_2_FC values and FDR values for two phz operons coding for phenazine biosynthesis (blue) and the genes *rhlABC* coding for rhamnolipid synthesis (red). Number of asterisks denotes FDR thresholds: ***, 0.001; **, 0.01; *, 0.05. (b) Box plots displaying the distributions of the abundance of representative metabolites of the phenazine and rhamnolipid class. Asterisks denote *P* values for comparison to the untreated controls (***, 0.001; **, 0.01; *, 0.05).

We observed a strong downregulation of both *phz1* and *phz2* operons for aminocoumarin-treated samples (Fig. 4a). Correspondingly, the concentrations of phenazine metabolites like phenazine-1-carboxylic acid and pyocyanin were strongly decreased (Fig. 4b and Table S2). Smaller or nonsignificant downregulating effects were exerted by fluoroquinolones and cystobactamids, respectively.

The rhamnolipids, glycolipids with surfactant-like properties, span another class of important virulence factors, because they enable P. aeruginosa to eliminate leucocytes and evade the most important immune mechanisms in the CF lung (13). The transcripts *rhlA*, *rhlB*, and *rhlC*, which encode key enzymes that attach the fatty acids to rhamnose units, were strongly downregulated upon aminocoumarin treatment (Fig. 4a). In line with this, decreased rhamnolipid concentrations were found in the metabolome (Fig. 4b). Thus, a differentiating property of the aminocoumarins, reflected on the transcriptome and metabolome level, lies in their strong downregulating effect on virulence factors.

### Cystobactamids induce a unique downregulation of a glucose catabolism pathway.

**FIG 5:**
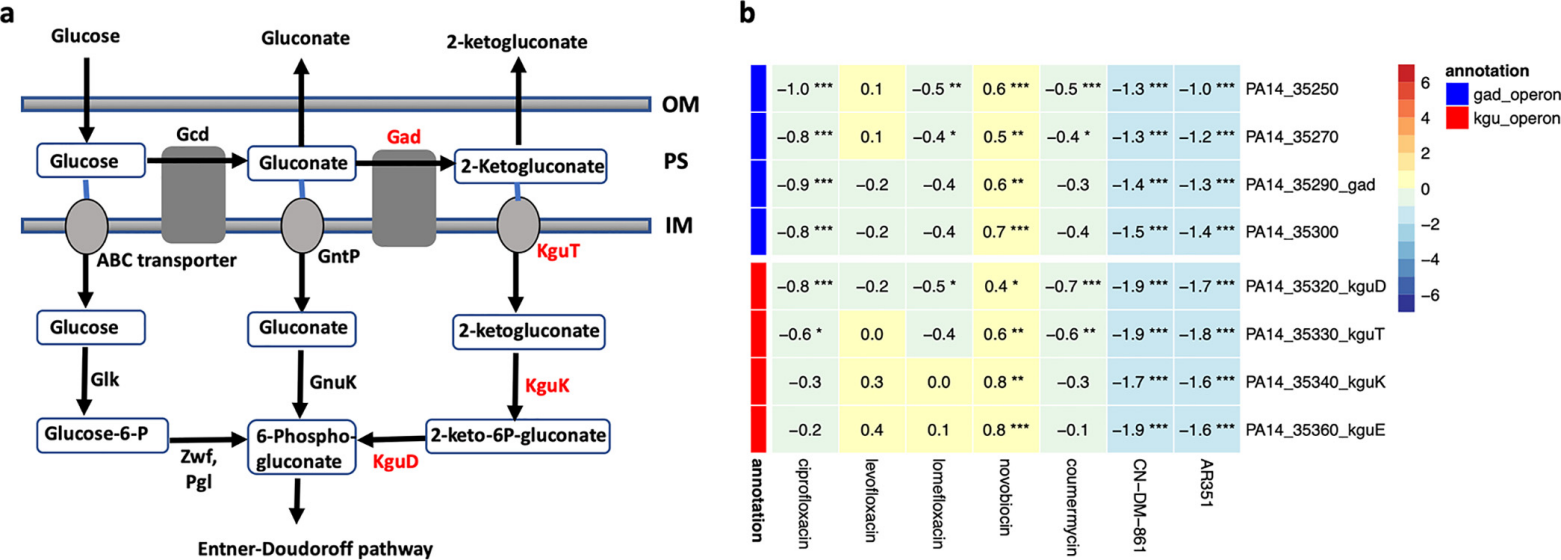
(a) Scheme of glucose utilization pathways in Pseudomonas subspecies, adapted from references 73 and 74. OM, outer membrane; PS, periplasmic space; IM, inner membrane; Gcd, glucose dehydrogenase; Gad, gluconate dehydrogenase; GntP, gluconate transporter; KguT, 2-ketogluconate transporter; Glk, glucokinase; GnuK, gluconate kinase; KguK, 2-ketogluconate kinase; Zwf, glucose-6-phosphate dehydrogenase; Pgl, 6-phosphogluconolactonase; KguD, 2-keto-6-phosphogluconate reductase. Downregulated enzymes are shown in red. (b) Heatmap displaying log_2_FC values and FDR values for the 2-ketogluconate (kgu) utilization operon (42) and the *gad* operon coding for the enzymes involved in transport and conversion of 2-ketogluconate into 6-phosphogluconate, which is then funneled into the Entner-Doudoroff pathway as shown in panel a (43). Number of stars denote FDR thresholds: ***, 0.001; **, 0.01; *, 0.05.

The only consistently downregulated pathway we found for the cystobactamids concerns two operons that were not fully annotated in the PA14 genome but could be matched to the 2-ketogluconate utilization operon of PAO1 (42) and the *gad* (gluconate dehydrogenase) operon (43) (Fig. 5).

P. aeruginosa metabolizes glucose exclusively via the Entner-Doudoroff pathway (44). Glucose enters the periplasm via an OprB porin and is then metabolized via three parallel routes that all converge on 6-phosphogluconate (45) (Fig. 5a). One of the routes comprises the 2-fold oxidation of glucose to gluconate and further to 2-ketogluconate in the periplasm, the transport of 2-ketogluconate to the cytoplasm, its phosphorylation by KguK to 2-keto-6-phophogluconate, and a final reduction to 6-phosphogluconate, which enters the Entner-Doudoroff pathway. This branch was selectively downregulated by cystobactamid treatment, whereas fluoroquinolones or aminocoumarins induced weak or nonsignificant effects. However, it is unclear whether addressing this arm of primary metabolism has an impact on the overall glucose metabolism of P. aeruginosa PA14. For P. aeruginosa PAO1, it has recently been shown by flux analysis approaches that approximately 90% of its glucose is oxidized into gluconate via the periplasmic route (46). Strikingly, the porin OprB, which mediates glucose uptake across the outer membrane, was strongly downregulated for the fluoroquinolones (2- to 4-fold), less (1.6- to 2.6-fold) for the aminocoumarins, and only 1.3- to 1.7-fold for the cystobactamids. In the case of the cystobactamids, glucose utilization was additionally diminished by downregulating enzymes of the 2-ketogluconate utilization operon, whereas for the other two classes, this effect was achieved by regulating OprB alone.

### General discussion.

Efforts to use untargeted metabolomics for mode-of-action studies of bioactive compounds rely on the assumption that molecules addressing a given target induce similar molecular signatures. The current study puts a note of caution on this assumption by demonstrating that there are clear differences depending on the drug class. Unsurprisingly, these were pronounced when different subunits with distinct functions, i.e., gyrase A versus gyrase B, were targeted. But even compounds addressing proximal binding sites on the same subunit like fluoroquinolones and cystobactamids could be differentiated by the transcriptomic, and to a lesser extend metabolomic, changes they induce in Pseudomonas aeruginosa PA14. A part of the discrimination can be attributed to differing sizes of effects going to the same direction, with some treatments falling above cutoff criteria and others below, whereas strong and significant effects going into opposite directions are rare. Thus, the cellular effects of different gyrase inhibitors are not fundamentally different. However, the findings have implications for mode-of-action assignments based on correlation methods. Ideally, more than one drug class for given targets or mechanisms is required to properly validate an inter- versus intratarget spread of signatures.

In this gyrase study, the aminocoumarins stand out by exerting the broadest effect and inducing the highest number of differentially regulated genes/metabolite features. We noted their strong downregulation of phenazines and rhamnolipids, important virulence factors of P. aeruginosa. The pronounced anti-virulence activity even at subinhibitory concentrations suggests a potential role for aminocoumarins as pathogenicity blockers of P. aeruginosa infections beyond that of a classical antibiotic, in particular when combined with compounds that exert synergism in PA14 like chlorhexidine or colistin (47).

Exposure to aminocoumarins also led to a surprisingly strong SOS response, which has been a hallmark of gyrase A poisoning. This observation contrasts a study in Staphylococcus aureus that describes an inhibiting effect of novobiocin at higher, not subinhibitory, concentrations on the ciprofloxacin-induced SOS response (48). On the other hand, the activation of the SOS response has been observed in Escherichia coli (49) and in Bacillus subtilis (50) following aminocoumarin treatment. We speculate that reactive oxygen species, indicated by an upregulation of stress response genes, are mediating DNA damage and subsequent induction of the SOS response.

The fact that aminocoumarins induce a much higher number of differentially expressed genes could be explained by the different inhibition mechanism of the gyrase. The DNA gyrase introduces negative supercoils in an ATP-dependent manner to maintain the DNA in an underwound state (51). By blocking the ATP-binding site, aminocoumarins cause partial relaxation toward a relaxed state (51). It has been shown that gene expression is directly influenced by the degree of supercoiling, with some genes showing enhanced expression when DNA is relaxed, while others are more highly expressed when negative supercoils are introduced (51, 52). Several studies demonstrate an effect of supercoiling on transcription initiation and detect bacterial genes that exhibit changes in expression that correlate with changes in DNA supercoiling (51–53). Increased amounts of exposed single-stranded DNA (ssDNA) as a consequence of negative supercoiling induced by the aminocoumarins provide an alternative explanation for the triggering of the SOS response (54). The binding of RecA to ssDNA leads to an increased proteolytic cleavage of LexA by RecA, thereby inducing expression of the LexA regulon (55).

A potential contributor to the observed differences between classes is off-target effects. Network-based approaches led to the finding that an average number of target proteins per drug of 6.3 can be calculated (56). Chemical proteomics approaches have revealed that it is also not uncommon to find multiple targets for antibiotics (57). For instance, vancomycin was found to inhibit the major staphylococcal autolysin Atl in addition to the primary mechanism of action exerted by binding to the dipeptide termini of peptidoglycan (58). Novobiocin inhibited the eukaryotic heat shock protein HSP90 by interacting with an ATPase binding domain (59), and an inhibition of bacterial ATPases might in principle contribute to the unique aminocoumarin signature observed here. The risk of hitting off-targets was deemed to be particularly high for novobiocin, because the applied IC_50_ was substantially higher than those of the other antibiotics. However, the number of regulated genes and features was actually 2-fold lower than that for coumermycin, which was applied at an IC_50_ in the low μM range, comparable to the other substances.

A striking finding of our study concerns the strong induction of pyocin production, ultimately leading to auto cell lysis, as demonstrated by the detection of eDNA. Pyocin upregulation in P. aeruginosa has been detected before, after triggering by hydrogen peroxide, ciprofloxacin, or genotoxic agents such as mitomycin C (32, 36–38, 60, 61). We show that the level of pyocin induction depends very much on the inhibitor class, with the fluoroquinolones inducing the strongest effects, and that the release of eDNA correlates directly with the endolysin expression. Evolutionarily, this altruistic suicide (36) of a bacterial subpopulation only makes sense by providing a survival advantage for the whole population, e.g., by releasing eDNA and enhancing biofilm formation (35). In fact, biofilm formation has been shown to drastically impair the efficacy of antibiotics such as fluoroquinolones (62). While this caveat concerns all compounds studied here, it remains to be probed whether the less pronounced pyocin induction of cystobactamids may translate to advantages in terms of biofilm formation.

### Conclusions.

Through a combination of transcriptomics and metabolomics, we demonstrated that the molecular signatures induced by three classes of gyrase inhibitors differ from each other. The findings enhance our understanding on the mode of actions of these important current (fluoroquinolones, aminocoumarins) and potential future (cystobactamids) antibiotics. They also suggest that signature correlation methods for target identification should consider intra-target-class variances. The differential effect of the three gyrase inhibitor classes is likely being caused by a combination of on- and off-target effects. To tease these effects apart, we plan to use resistant mutants of PA14 with strongly reduced target affinity. Another logical expansion of the approach to further gyrase A and B inhibitor classes, or to other prominent antibiotic targets (e.g., protein translation, cell wall synthesis), is also subject to future studies.

## MATERIALS AND METHODS

### Reagents.

Cystobactamids CN-DM-861 and AR351 were synthesized in the lab of the corresponding author according to reference 21. Ciprofloxacin was purchased from AppliChem. Coumermycin A1, levofloxacin, lomefloxacin HCl, novobiocin sodium salt, trimethoprim, nortriptyline HCl, caffeine, (NH_4_)_2_SO_4_, and Fe(II)SO_4_ 7×H_2_O were obtained from Sigma-Aldrich. Glipizide was purchased from ACROS Organics, and (S)-naproxen was purchased from Cayman Chemical Company. d(+)-Glucose monohydrate and K_2_HPO_4_ were obtained from Merck Millipore, and MgSO_4_ 7×H_2_O and casamino acid were obtained from Roth. Acetonitrile ultra LC/MS grade, water ultra LC/MS grade, and methanol ultra LC/MS grade were obtained from Fisher Scientific.

### Bacteria.

The Pseudomonas aeruginosa PA14 strain was obtained from the DSMZ (DSM 19882).

### MIC and IC_50_ determination via microdilution method.

MIC values were determined following the microdilution method but using BM2 medium instead of Mueller-Hinton broth. A starting OD of 0.1 of Pseudomonas aeruginosa PA14 was used as inoculum in BM2 medium. Cultivation at 37°C was carried out in microtiter plates.

Compounds were serially diluted in 96-well microtiter plates in triplicate. After incubation of the plates for 18 h at 37°C, the absorbance at 600 nm was measured to determine the MIC value and the IC_50_ value. We used a Gompertz function to fit the data in R and determine the IC_50_ and MIC according to Lambert and Pearson (63).

### Bacterial culture and addition of gyrase inhibitors.

For profiling of the effects of gyrase inhibitors on the Pseudomonas aeruginosa PA14 metabolome and transcriptome, planktonic bacteria were cultivated to mid-exponential phase (OD_600_ = 0.6) in the presence of subinhibitory concentration of the antibiotic.

A preculture was prepared by inoculation of 20 ml BM2 medium [2 mM (NH_4_)_2_SO_4_, 40 mM K_2_HPO_4_, 22 mM KH_2_PO_4_, 2 mM MgSO_4_, 10 μM FeSO_4_, 0.4% (wt/vol) glucose, 0.01% (wt/vol) Casamino Acids] with Pseudomonas aeruginosa PA14 from the glycerol stock. The preculture was shaken (150 rpm) overnight at 37°C. The 1:10 diluted preculture was then used to calculate the precise dilution to inoculate a 20 ml BM2 culture at an OD_600_ of 0.05. Immediately after adjusting the starting OD, the amount of gyrase inhibitor that resulted in the concentration corresponding to the IC_50_ that was determined via the broth microdilution method was added to the culture (see Table 1).

The compounds were added from a dimethyl sulfoxide (DMSO) stock solution, apart from lomefloxacin and novobiocin that were added from an H_2_O stock solution. For each flask it was ensured that the same amount of DMSO was added, so in the case of lomefloxacin and novobiocin, 10.1 μl of DMSO was additionally added, and for the untreated control we used a mock treatment of 10.1 μl DMSO as well. For each gyrase inhibitor that was tested and for the control, three independent 20 ml main culture flasks were prepared and incubated at 37°C and 150 rpm until an OD of 0.6 was reached. The OD was monitored regularly and growth curves were recorded. At the target OD of 0.6, 2 ml of culture was transferred to an Eppendorf tube and centrifuged for 5 min (9,000 × *g*, 4°C) for metabolomics measurements. The supernatant was discarded, and the pellet was washed with 0.9% NaCl and frozen with liquid nitrogen. For RNA-Seq measurements of the same sample, 0.75 ml from the same culture (at OD of 0.6) was added to an Eppendorf tube with 0.75 ml RNAprotect (Qiagen, Hilden, Germany), briefly agitated, and left for 10 min at room temperature. After centrifugation at 8,000 rpm, the supernatant was removed and frozen with liquid nitrogen.

### Measurements of extracellular DNA in planktonic culture.

Culture samples of P. aeruginosa PA14 were cultivated and treated with ciprofloxacin, novobiocin, or CN-DM-861 as described in the section above.

To quantify the amount of extracellular DNA (eDNA) in planktonic cell culture, we employed the method described by Allesen-Holm et al. (38). After reaching the target OD of 0.6, cultures were centrifuged (3 min, 10,000 rpm) and the supernatant was transferred to a new Eppendorf tube. NaCl solution (2.5 M) was added to the supernatant to a concentration of 0.25 M. After vortexing, a 2:1 volume of ethanol was added to precipitate the DNA. After vortexing and standing for 5 min, the suspension was centrifuged (10 min, 16,000 rpm) and the supernatant was removed with a pipette. The residue (eDNA) was dissolved in Tris-EDTA (TE) buffer and thoroughly vortexed. The amount of eDNA was quantified by measuring the OD_260_ and using TE buffer as a reference. Four independent cultures (biological replicates) were used for each treatment.

### Metabolomics extraction and LC-MS analysis.

For the analysis of the intracellular metabolome, cell pellets were extracted with 500 μl 80% methanol containing caffeine (0.1 mg/liter), nortriptyline (0.1 mg/liter), glipizide (0.3 mg/liter), and naproxen (0.8 mg/liter) as internal standards. Extraction was achieved through shaking vigorously for 5 min and sonication in an ultrasonic bath for 20 min at 0°C. After centrifugation (13,000 rpm, 4°C), 400 μl of the supernatant was concentrated to dryness using a SpeedVac and resuspended in 40 μl acetonitrile/water (1:1, containing 0.1% [vol/vol] formic acid) using ultrasonication for dissolving the residue.

For each sample, 1 μl was analyzed by reversed phase ultrahigh-performance liquid chromatography coupled to quadrupole time-of-flight mass spectrometry. In addition, a pool sample was prepared by mixing 5 μl of each sample.

The samples were separated using ultra high-performance liquid chromatography, performed on a Dionex Ultimate 3000 UPLC system (Thermo Fisher Scientific, Waltham, MA) using a 150 by 2.1 mm Kinetex C_18_ column with 1.7-μm particle size (Phenomenex, Aschaffenburg, Germany) column with a flow rate of 300 μl/min.

Gradient elution with water with 0.1% (vol/vol) formic acid as eluent A and acetonitrile with 0.1% (vol/vol) formic acid as eluent B was run as follows: 1% B for *t* = 0 min to *t* = 2 min, linear gradient from 1% B to 100% B from *t* = 2 min to *t* = 20 min, hold 100% B until *t* = 25 min, and linear gradient from 100% B to 1% B from *t* = 25 min to *t* = 30 min.

The samples were analyzed by positive mode electrospray ionization quadrupole time-of-flight mass spectrometry on a maXis HD QTOF (Bruker, Bremen, Germany) in full scan mode (50 to 1,500 Da). Accurate masses were obtained by internal calibration using an ion cluster of sodium formate and lock mass calibration. The pooled sample was analyzed using data-dependent MS/MS by collision-induced dissociation of the three most abundant ions in each scan, making use of Bruker’s “Smart Exclusion” functionality to minimize multiple fragmentation of the same ion.

### RNA isolation and library preparation and sequencing.

The bacterial suspensions treated with RNAprotect (Qiagen) were stored at −80°C. Libraries for transcriptomics were generated according to references 64 and 65.

RNA samples were quality checked by the use of the RNA Nano kit with an Agilent Bioanalyzer 2100 (Agilent Technologies, CA, USA). To remove rRNA, the Ribo-Zero bacteria kit (Illumina, CA, USA) was used. Samples were sequenced in paired-end mode on an Illumina NovaSeq 6000 device (2 × 50 bp).

### Bioinformatics processing of metabolomics data.

LC-MS data generated with the Bruker maXis HD mass spectrometer were converted to the mzXML format from the Bruker centroid data using Bruker Compass Xport. Preprocessing of the mzXML raw files was carried out using XCMS (version 3.4.4) (66) as follows.

For peak picking, the centWave algorithm was employed with parameter settings peakwith from 5 to 25 s, ppm = 10, snthresh = 100, mzdiff = 0.01, prefilter = c(2, 1000), noise = 100. For chromatogram alignment, the obiwarp algorithm was used with default settings, and grouping of peaks from different samples was achieved by using the group.density function with settings bw = 5, mzwid = 0.015, minfrac = 0.5, minsamp = 1.

Feature annotation (isotopic features and adduct formations, dimers, multimers, neutral losses) was carried out using the CAMERA package (version 1.38.1) (67) with parameters perfwhm = 0.6, mzabs = 0.01, cor_eic_th = 0.75.

Further processing of the peak table employed an in-house script involving the following steps. A retention time filter (features with a retention time lower than 50 s or higher than 1,200 s were removed from the peak table) was employed to get rid of the sodium formate cluster peak and the signals arising from impurities that elute during washing of the column with 100% acetonitrile. To account for experimental variability, peak areas were normalized using the internal standard nortriptyline. After removal of all features belonging to internal standards, the data for each sample were further normalized through the use of the OD at harvest of the respective culture as a proxy for cell number. Missing value imputation was carried out using the imputeRowMinRand function of the XCMS package, which replaces missing values with random numbers from a normal distribution based on the row minimum. For metabolite identification, tandem mass spectra recorded from a pooled sample were compared to an in-house database of spectra from authentic standards and/or metabolite databases using the Bruker DataAnalysis software (version 4.0, for results see Tables S1 and S3). To perform hierarchical clustering, the data set was scaled, Euclidean distance was utilized as distance measure, and Ward’s method was utilized as linkage criterion. For PCA, the data set was scaled and centered. To identify significantly abundant features, Welch’s *t* test was used and a threshold of log_2_(fold change) of log_2_(1.5) or greater and of −log_2_(1.5) or lower, respectively, with a *P* value of <0.05 was applied. The raw data are deposited as MassIVE data set MSV000086820 (https://massive.ucsd.edu/). The R scripts used for preprocessing and analyzing the data are deposited in a GitHub archive: https://github.com/raimofranke/PA14_gyr_inhibitors.

### Bioinformatics processing of transcriptomics data.

Short reads were aligned to the UCBPP-PA14 reference genome (NC_008463, available for download from the Pseudomonas genome database, http://pseudomonas.com) using the subread algorithm (68) implemented in R (R version 3.5.3, Foundation for Statistical Computing, Vienna, Austria; Rsubread version 1.32.4) (69). Count estimation was performed using the featureCounts function (70) as implemented in Rsubread. Differential gene expression analysis was performed using the R package edgeR (version 3.24.3) (71). Trimmed mean of M-values (TMM) normalization was performed, followed by quasi-likelihood F-test to test for significant differential expression in each gene (glmQLFTest-function). To correct for multiple testing, the Benjamini Hochberg procedure was used to calculate the false discovery rate (FDR). For the identification of differentially expressed genes between gyrase inhibitor treatment and untreated control, a threshold of log_2_(fold change) of 1 or greater and of −1 or lower, respectively, with FDR of <0.05 was applied. For clustering and PCA, moderated log counts per million (log CPM) were calculated using the CPM function. For PCA, centered log CPM data were used. For hierarchical clustering, the log CPM data were used with Euclidean distance as distance measure and Ward’s method as linkage criterion. For annotation of the results table (Table S2) with KEGG pathway, GO term, and pseudoCAP class information, the annotator functionality of the BACTOME database was used (29).

### Data and code availability.

The RNA-Seq data generated during this study have been deposited in NCBI’s Gene Expression Omnibus (72) and are accessible through GEO series accession number GSE166602. The metabolomics data have been deposited in the MassIVE database (https://massive.ucsd.edu/) under accession number MSV000086820. The R scripts used for processing the RNA-Seq, metabolomics data, and generation of figures have been deposited in a GitHub archive: https://github.com/raimofranke/PA14_gyr_inhibitors.
