# Supplementary material for: Targeting Bacterial Gyrase with Cystobactamid, Fluoroquinolone, and Aminocoumarin Antibiotics Induces Distinct Molecular Signatures in Pseudomonas aeruginosa
Source: mSystems. 2021 Jul 13;6(4):e00610-21. doi: 10.1128/mSystems.00610-21 (PMC8407119; doi:10.1128/mSystems.00610-21)
Supplement: TABLE S1 [file msystems.00610-21-st001.docx]

| **m/z** | **RT [s]** | **Annotation** | **Metabolite** | **ID method** | **ID level** |
| --- | --- | --- | --- | --- | --- |
| 146.165 | 61.8 | spermidine | spermidine | MS, MS2, RT | 1 |
| 89.107 | 64.8 | putrescine | putrescine | MS, RT | 1 |
| 148.060 | 73.6 | glutamic acid | glutamic acid | MS, MS2, RT | 1 |
| 244.079 | 76.2 | N-acetyl glucosamine/mannosamine putative [M+Na]+ | N-acetyl glucosamine/mannosamine putative | MS | 3 |
| 116.070 | 77.1 | proline | proline | MS, RT | 1 |
| 324.059 | 78.7 | CMP | Cytidine-5´-monophosphate | MS, MS2, RT | 1 |
| 277.103 | 78.7 | GluGlu | Glu-Glu | MS, MS2 | 2 |
| 118.086 | 84.6 | betaine | betaine | MS, RT | 1 |
| 169.035 | 86.0 | uric acid | uric acid | MS, RT | 1 |
| 332.562 | 86.0 | NAD [M+2]2+ | NAD | MS, RT | 1 |
| 348.070 | 86.0 | adenosien-5´-monophosphate | adenosien-5´-monophosphate | MS, MS2, RT | 1 |
| 150.058 | 86.2 | methionine | methionine | MS, RT | 1 |
| 132.102 | 122.4 | leucine/isoleucine/norleucine | leucine/isoleucine/norleucine | MS, MS2, RT | 1 |
| 182.081 | 126.2 | tyrosine | tyrosine | MS, MS2, RT | 1 |
| 140.034 | 174.5 | 6-hydroxy nicotinic acid | 6-hydroxy nicotinic acid | MS, RT | 1 |
| 166.086 | 210.1 | phenylalanine | phenylalanine | MS, MS2, RT | 1 |
| 205.097 | 363.2 | tryptophan | tryptophan | MS, RT | 1 |
| 211.086 | 392.0 | pyocyanin | pyocyanin | MS, MS2, RT | 1 |
| 162.055 | 507.9 | DHQ | DHQ | MS, RT | 1 |
| 243.087 | 557.7 | lumichrome | lumichrome | MS, RT | 1 |
| 224.081 | 641.2 | phenazine-1-carboxamide | phenazine-1-carboxamide | MS, RT | 1 |
| 225.066 | 690.2 | phenazine-1-carboxylic acid | phenazine-1-carboxylic acid | MS, MS2, RT | 1 |
| 258.149 | 767.1 | C7:1-QNO | C7:1-QNO | MS, MS2 | 2 |
| 242.154 | 786.6 | C7:1-HQ | C7:1-HQ | MS, MS2 | 2 |
| 244.170 | 790.1 | HHQ | HHQ | MS, MS2, RT | 1 |
| 260.165 | 796.3 | C7-QNO | C7-QNO | MS, MS2 | 2 |
| 297.242 | 833.8 | Dihydroxyoctadecenoic acid  [M-H2O +H]+ | Dihydroxyoctadecenoic acid | MS, MS2 | 2 |
| 258.185 | 846.5 | C8-HQ | C8-HQ | MS, MS2 | 2 |
| 274.180 | 848.9 | C8-QNO | C8-QNO | MS, MS2 | 2 |
| 286.180 | 858.2 | C9:1-QNO (I) | C9:1-QNO | MS, MS2 | 2 |
| 288.187 | 871.3 | C9-QNO (I) | C9-QNO | MS, MS2 | 2 |
| 286.180 | 871.4 | C9:1-QNO (II) | C9:1-QNO | MS, MS2 | 2 |
| 284.201 | 894.7 | C10:1-HQ | C10:1-HQ | MS, MS2 | 2 |
| 270.185 | 900.1 | C9:1-HQ | C9:1-HQ | MS, MS2 | 2 |
| 288.196 | 900.9 | C9-QNO (II) | C9-QNO | MS, MS5 | 2 |
| 272.201 | 900.9 | C9-HQ | C9-HQ | MS, MS7 | 2 |
| 452.277 | 911.9 | LPE(16:1) (I) | LPE(16:1) | MS, MS2 | 2 |
| 452.277 | 929.8 | LPE(16:1) (II) | LPE(16:1) | MS, MS2 | 2 |
| 314.211 | 938.9 | C11:1-QNO (I) | C11:1-QNO | MS, MS2 | 2 |
| 298.217 | 939.4 | C11:1-HQ (I) | C11:1-HQ | MS, MS2 | 2 |
| 319.224 | 946.8 | Hydroxyoctadecadienoic acid (I) [M+Na]+ | Hydroxyoctadecadienoic acid | MS, MS2 | 1 |
| 673.376 | 957.1 | Rha-Rha-C10-C10 [M+Na]+ | Rha-Rha-C10-C10 | MS, MS2 | 2 |
| 314.212 | 971.2 | C11:1-QNO (II) | C11:1-QNO | MS, MS2 | 2 |
| 328.227 | 986.7 | C12:1-QNO | C12:1-QNO | MS, MS2 | 2 |
| 454.293 | 992.0 | LPE(16:0) (I) | LPE(16:0) | MS, MS2 | 2 |
| 297.242 | 1000.6 | Hydroxyoctadecadienoic acid (II) | Hydroxyoctadecadienoic acid | MS, MS2 | 2 |
| 316.227 | 1002.8 | C11-QNO | C11-QNO | MS, MS2 | 2 |
| 699.392 | 1003.1 | Rha-Rha-C10-C12:1[M+Na]+ | Rha-Rha-C10-C12:1 | MS, MS2 | 2 |
| 527.319 | 1004.3 | Rha-C10-C10 [M+Na]+ | Rha-C10-C10 | MS, MS2 | 2 |
| 298.217 | 1006.7 | C11:1-HQ (II) | C11:1-HQ | MS, MS2 | 2 |
| 300.232 | 1007.4 | C11-HQ | C11-HQ | MS, MS2 | 2 |
| 454.293 | 1016.9 | LPE(16:0) (II) | LPE(16:0) | MS, MS2 | 2 |
| 342.243 | 1027.6 | C13:1-PQS | C13:1-PQS | MS, MS2 | 2 |
| 326.248 | 1032.4 | C13:1-HQ (I) | C13:1-HQ | MS, MS2 | 2 |
| 324.232 | 1032.9 | C13:2-HQ | C13:2-HQ | MS, MS2 | 2 |
| 701.408 | 1038.5 | Rha-Rha-C10-C12 [M+Na]+ | Rha-Rha-C10-C12 | MS, MS2 | 2 |
| 281.247 | 1039.1 | PUFA(18:2) | PUFA(18:2) | MS, MS2 | 2 |
| 480.308 | 1039.6 | LPE(18:1) (I) | LPE(18:1) | MS, MS2 | 2 |
| 553.334 | 1051.4 | Rha-C10-C12:1 [M+Na]+ | Rha-C10-C12:1 | MS, MS2 | 2 |
| 480.308 | 1061.9 | LPE(18:1) (II) | LPE(18:1) | MS, MS2 | 2 |
| 312.195 | 1063.7 | C11:2-QNO | C11:2-QNO | MS, MS2 | 2 |
| 555.350 | 1087.4 | Rha-C10-C12 | Rha-C10-C12 | MS, MS2 | 2 |
| 326.248 | 1111.7 | C13:1-HQ (II) | C13:1-HQ | MS, MS2 | 2 |
| 370.274 | 1121.0 | C15:1-QNO | C15:1-QNO | MS, MS2 | 2 |
| 255.232 | 1124.8 | palmitoleic acid | palmitoleic acid | MS, MS2, RT | 1 |
| 260.164 | 1192.5 | PQS | PQS | MS, MS2 | 1 |
| 283.263 | 1192.8 | oleic acid | oleic acid | MS, MS2, RT | 1 |
