# Supplementary figures and images for: Targeting Bacterial Gyrase with Cystobactamid, Fluoroquinolone, and Aminocoumarin Antibiotics Induces Distinct Molecular Signatures in Pseudomonas aeruginosa
Source: mSystems. 2021 Jul 13;6(4):e00610-21. doi: 10.1128/mSystems.00610-21 (PMC8407119; doi:10.1128/mSystems.00610-21)

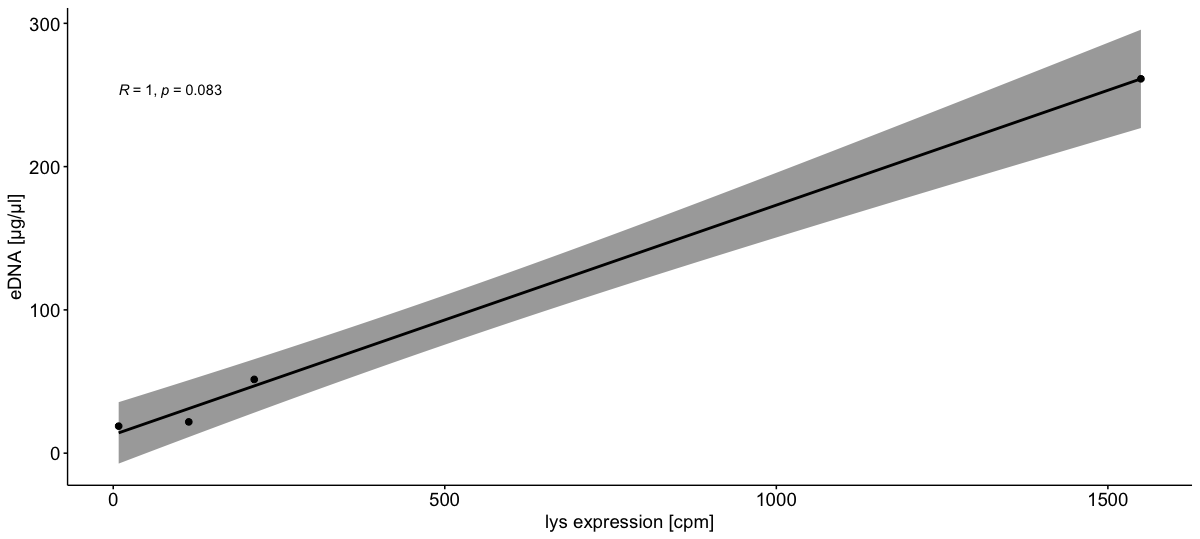

Supplement: FIG S1 [file msystems.00610-21-sf001.tif]
